# New insights into the evolutionary rate of HIV-1 at the within-host and epidemiological levels

**DOI:** 10.1098/rspb.2012.0595

**Published:** 2012-05-16

**Authors:** Katrina A. Lythgoe, Christophe Fraser

**Affiliations:** Department of Infectious Disease Epidemiology, School of Public Health, Imperial College London, St Mary's Campus, London W2 1PG, UK

**Keywords:** HIV, mathematical model, rate of evolution, viral latency

## Abstract

Over calendar time, HIV-1 evolves considerably faster within individuals than it does at the epidemic level. This is a surprising observation since, from basic population genetic theory, we would expect the genetic substitution rate to be similar across different levels of biological organization. Three different mechanisms could potentially cause the observed mismatch in phylogenetic rates of divergence: temporal changes in selection pressure during the course of infection; frequent reversion of adaptive mutations after transmission; and the storage of the virus in the body followed by the preferential transmission of stored ancestral virus. We evaluate each of these mechanisms to determine whether they are likely to make a major contribution to the mismatch in phylogenetic rates. We conclude that the cycling of the virus through very long-lived memory CD4^+^ T cells, a process that we call ‘store and retrieve’, is probably the major contributing factor to the rate mismatch. The preferential transmission of ancestral virus needs to be integrated into evolutionary models if we are to accurately predict the evolution of immune escape, drug resistance and virulence in HIV-1 at the population level. Moreover, early infection viruses should be the major target for vaccine design, because these are the viral strains primarily involved in transmission.

## 1. Introduction

Owing to its short-generation time and error-prone replication, the HIV genome evolves at incredible rates within hosts [1,2]. However, there is growing evidence that, over calendar time, HIV accumulates mutations at a considerably reduced rate (about 2× to 6× slower) at the between-host (epidemic) level than expected, given what we know about its rate of evolution within hosts [3–6]. This is surprising because there is no obvious reason why the virus's molecular clock should tick slower at the between-host level. Three mechanisms have been suggested that could result in the mismatch in phylogenetic rates of divergence (‘rate mismatch’ hereafter), which we have termed ‘stage-specific selection’, ‘adapt and revert’, and ‘store and retrieve’ [6–8].

First, under stage-specific selection, it is argued that the rate mismatch occurs because selection is weaker in early infection, resulting in a lower rate of diversification per unit time when measured from the time of infection to transmission (the period that determines between-host rates of divergence), than when measured during chronic infection (the period during which within-host rates of divergence are measured). In the absence of stage-specific selection, we would expect the rate of evolution per unit time to be independent of when transmission occurs. If transmission tends to occur during early infection, the rate of divergence per transmission event will be slower than if transmission tends to occur late, but when measured over calendar time, the two rates will be the same.

Second, under adapt and revert, it is argued that mutations that are adaptive in one individual are likely to be maladaptive in another owing to, for example, different human leukocyte antigen (HLA) backgrounds, and thus will revert after transmission. If a sufficient proportion of mutations that are fixed within an infected host revert once a new host is infected, then a mismatch in phylogenetic rates is likely to emerge because not all mutations accumulating at the within-host level will accumulate at the between-host level.

Finally, under store and retrieve, it is argued that ancestral sequences (i.e. those that are more similar to the infecting viral strain than to contemporary circulating virus strains within the host) are stored in the body and are preferentially transmitted, resulting in faster rates of divergence when measured at the within-host level compared with the between-host level. Preferential transmission of ancestral strains could occur either because ancestral strains have an intrinsic transmission advantage, or because virus is more likely to be stored in the genital tract, thus leading to preferential transmission during sexual transmission.

Our aim is to establish which, if any, of these mechanisms are likely to make major contributions to the observed mismatch in phylogenetic rates, or whether additional or alternative mechanisms are required. We select among plausible mechanisms by a process of elimination. By comparing previously published estimates of the rate of synonymous and non-synonymous mutations at the within- and between-host levels, we argue that neither stage-specific selection nor adapt and revert is likely to explain a substantial proportion of the mismatch, especially among synonymous mutations. Of the mechanisms we consider, store and retrieve is the only mechanism likely to make a major contribution to the mismatch in phylogenetic rates. Moreover, by comparing rates of divergence between virus circulating in different host populations, we argue that stored ancestral virus is probably preferentially transmitted because it has an inherent transmission advantage, rather than because virus is more likely to be stored in the genital tract than in other parts of the body.

To test whether the store and retrieve mechanism, with an intrinsic transmission advantage to ancestral virus, is sufficient to quantitatively explain the observed mismatch in phylogenetic rates, we develop a simple mathematical model of within-host HIV evolution coupled to store and retrieve transmission dynamics. We find that if virus is stored in latent form in long-lived cells for a sufficient amount of time, in the order of years, then the store and retrieve mechanism can explain the mismatch in phylogenetic rates of divergence. We thus conclude that the store and retrieve model of viral evolution can qualitatively and quantitatively explain observed trends, and we explore some evolutionary and public health consequences of these findings.

## 2. Evidence for a mismatch in phylogenetic rates

To estimate rates of divergence at the within- and between-host levels, viral sequences taken at different time points are compared, either from the same patient during the course of an infection (to calculate within-host rates) or from different patients during the course of an epidemic (to calculate between-host rates). In general, it is difficult to compare estimates of the rate of divergence of HIV-1 at the within- and between-host levels because of differences in study design, such as the use of different HIV-1 subtypes, different host populations, different segments of the viral genome, different alignment techniques and different statistical methods. However, where authors have controlled for these differences a strong rate mismatch has been observed, with a 2× to 6× faster rate of divergence for *env* at the within-host than at the between-host level in populations where the virus is transmitted sexually [5,6]. Moreover, we can use published data to separately compare rates of divergence for synonymous and non-synonymous mutations within *env* at the within- and between-host levels ([3,4] and table 1), noting, in particular, that the mismatch in phylogenetic rates is of a similar magnitude for both types of mutations.

**Table 1.** Comparison of within- and between-host rates of divergence. All estimates are for subtype B.

	synonymous (substitutions per site per year)	non-synonymous (substitutions per site per year)	reference
within- host^a,b^	5.5 × 10^−3^	9.45 × 10^−3^	[4]
between-host^c^	1.3 × 10^−3^	3.4 × 10^−3^	[3]

^a^*env* position 7026–7616 relative to HXB2 (Philippe Lemey, personal communication).

^b^An average of the internal branch rates was taken for the moderate and slow progressors reported in Lemey *et al*. [4] so as to make the values directly comparable with Abecasis *et al*. [3].

^c^*env* position 6500–7500 relative to HXB2 (Philippe Lemey 2011, personal communication).

Further evidence for a rate mismatch comes from the Rakai Community Cohort Study, where the chain of transmission of HIV-1 from one partner to another, and importantly the timing of transmission events, is often known. Here, it has been shown that for known transmission chains containing three people, the rate of divergence of gp41 in *env*, when measured among these three individuals, is only half the rate of divergence measured at the within-host level [9].

Preliminary analyses of whole viral genomes appear to confirm that the mismatch in rates is present for all genes, though may be greater for *env* than for other genes (Samuel Alizon 2011, personal communication).

## 3. Evaluating the possible mechanisms resulting in rate mismatch

### (a) Stage-specific selection

Stage-specific selection has been invoked as a mechanism explaining why, when measured over calendar time, viruses from similar clades evolve at very different rates in different epidemics [10]. In rapid epidemics of injecting drug users (IDUs), the virus was found to evolve four times slower than in slower generalized epidemics in sub-Saharan Africa. Maljkovic Berry *et al*. [10] hypothesized that this discrepancy arises because hosts do not mount an effective immune response immediately upon infection (this is what we have termed stage-specific selection). Among IDUs, the authors suggest that the virus is transmitted so fast from person to person that hosts do not have time to mount effective immune responses and thus drive the evolution of the virus. Subsequently, Pybus & Rambaut [6] put forward stage-specific selection as one of the main hypotheses to explain the difference in between-host and within-host evolutionary clock rates. They argue that if transmission tends to occur in early infection, the rate of evolution of the virus over calendar time will be slower at the between-host level than at the within-host level.

As a consequence of stage-specific selection, there will be a mismatch in the measured within- and between-host rates of diversification; the between-host rate of diversification is determined by the number of genetic substitutions accumulated by the viral population between the time of infection and the time of onward transmission (i.e. including the period during which selection is weak and adaptation slow), whereas the within-host rate of diversification tends to be measured only during chronic infection (i.e. when selection is strong and adaptation fast). If there is no stage-specific selection, then we would expect the evolutionary clock to tick at a similar rate regardless of whether transmission tends to occur early or late in infection because the clock is measuring divergence over calendar time, not per transmission.

Direct evidence for this proposition is equivocal. Studies following the evolution of the virus within-hosts do not show clear evidence of an early ‘eclipse’ phase of slow within-host viral evolution [1]. More recent studies, using sophisticated sequencing techniques, have found evidence of extremely rapid evolution driven by immune selection in the first months of infection [8,11–14]. As far as we are aware, only one patient has been followed into chronic infection, using similar techniques, and for this patient, the rate of evolution does not appear to be much higher in the chronic than in the acute phase [14]; however, because these results are for a single patient, the observation neither rules in nor rules out a general pattern of an even stronger selection in later infection.

However, because the mismatch in phylogenetic rates at the within- and between-host levels is observed for synonymous as well as non-synonymous mutations ([13] and table 1), it is unlikely that stage-specific selection is a main factor explaining the observed rate mismatch. Even accounting for hitchhiking effects [15], stage-specific selection is predicted to have a greater influence on non-synonymous mutations because it should influence the rate of accumulation of adaptive mutations to a much greater extent than neutral or nearly neutral mutations. This argument hinges on the assumption that synonymous mutations experience much weaker levels of selection than do non-synonymous mutations. In compact genomes such as HIV-1, it is likely that some synonymous mutations will be subject to selection owing their effect on, for example, the secondary structure of the RNA genome. However, we think it unlikely that synonymous mutations experience similar levels of selection to non-synonymous mutations: a new study has shown that for ssRNA viruses the selection effect on non-synonymous mutations is about five times greater than that on synonymous mutations [16]. Moreover, in HIV-1, synonymous mutations are likely to be non-neutral owing to their effects on the secondary structure of the RNA genome [17], but selection tends to constrain the rate of evolution at sites affecting secondary structure, rather than enhancing it [17,18], and, in addition, this effect is apparent for both synonymous and non-synonymous mutations [17].

It is also worth noting that the comparisons of the rates of divergence at the within- and between-host levels have focused on populations where the virus is sexually transmitted, and therefore, the effect of slow divergence during early infection would be likely to be swamped by adaptive processes occurring later on in infection, and cannot explain the mismatch that we reported in table 1. It would be interesting to compare rates of divergence of synonymous and non-synonymous mutations in populations among IDUs. However, for sexual transmission at least, we conclude that stage-specific selection has little influence on the rate mismatch.

### (b) Adapt and revert

After transmission to a new host, HIV-1 partially reverts towards a consensus wild-type sequence, though in a heterogeneous manner that is difficult to predict [7,19–21]. Reversion is unsurprising because newly infecting viruses find themselves in an environment in which the host-immune system is naïve to the virus and the recipient is likely to have a different HLA type to the donor. If most mutations that are fixed within an infected host are adaptive in that host, but revert once a new host is infected, then a mismatch in phylogenetic rates can emerge because not all mutations accumulating at the within-host level will accumulate at the between-host level.

Direct evidence of reversion is complex to interpret. Reversion appears to be fast for only the most costly adaptive mutations, which are rare, while other adaptive mutations revert slowly or not at all [19,20,22].

In terms of explaining the mismatch in rates summarized in table 1, as with stage-specific selection, we note that the rate mismatch is similar for non-synonymous and synonymous mutations, ruling out adapt and revert as a primary mechanism affecting the rate mismatch; adapt and revert should have a much greater affect on non-synonymous than synonymous mutations, which we do not observe. However, adapt and revert might still have a secondary role, perhaps explaining why the rate mismatch appears to be greater for *env* than for other genes.

### (c) Store and retrieve

Ancestral HIV sequences can be ‘stored’ within a host for long periods of time, creating within-host heterogeneity in the amount of evolution that viral lineages have undergone within a single host at any given moment in time [23,24]. HIV-1 replicates most productively when infecting active CD4^+^ T cells [25]. The double-stranded RNA virus enters the host cell, is reverse transcribed into cDNA and then integrated into the host genome, where it is known as provirus. This proviral DNA is then transcribed into RNA, and new virions are assembled that bud off from the host-cell membrane. This whole process takes about one to two days [2,26]. A smaller proportion (approx. 1%) of the virus is produced in a somewhat slower process, for example by replication in macrophages [27]. Occasionally, CD4^+^ T cells with integrated provirus will enter a resting phase; these latently infected memory T cells effectively store virus creating a very stable viral archive [28]. Months, or even years, after entering the resting phase, latently infected resting memory T cells can become reactivated, at which point the provirus is able to resume replication and the stored viral strain is retrieved from the archive [29–31]. In addition, there may be some additional and as yet unidentified long-lived viral reservoirs that also contribute to this process [32].

Irrespective of the exact mechanism of storage, if ancestral virus is preferentially transmitted, then the evolutionary clock is predicted to tick rapidly within-hosts, but at transmission, the hands of the clock are metaphorically turned back. Viral storage and retrieval through preferential transmission would lead to slower rates of divergence when measured at the population level.

Evidence is accumulating that ancestral virus is at least sometimes preferentially transmitted. By studying viruses in many transmitting couples, it has been shown that virus circulating in newly infected heterosexual recipients (within a year of infection) tends to be more closely related to donor ancestral sequences than contemporary sequences circulating within the donor at the time of infection [33], and that the HIV-1 sequence a person acquires through heterosexual transmission tends to be similar to the sequence that she/he transmits [9].

Recent evidence from high-resolution phylogenies is more equivocal; data from Herbeck *et al.* [8] clearly show the transmission of ancestral virus in one of three men who have sex with men transmission pairs, with one further pair being more difficult to interpret, and one transmission occurring during acute infection (and as such all viruses are similar to the ancestral strain). Of four transmission pairs studied in Li *et al*. [34], two were transmissions that occurred during acute infection, and in two further cases, it did not appear that archived virus was preferentially transmitted. Thus, we conclude that the evidence is limited, but points perhaps to a dichotomous process, where sometimes truly ancestral virus is transmitted, rewinding the evolutionary clock completely, and sometimes extant virus is transmitted, so that the evolutionary clock is not rewound at all. In our analyses later, we will focus on the average effect, as that is all we can measure with population samples, but this dichotomous model should be considered in further work.

A key prediction of the store and retrieve mechanism is that it should affect synonymous and non-synonymous sites equally. Therefore, the observation that the mismatch in phylogenetic rates affects synonymous and non-synonymous mutations to a similar extent provides strong support that store and retrieve is a major mechanism affecting the rate mismatch.

For store and retrieve to result in a mismatch in phylogenetic rates, there must be a mechanism allowing for the preferential transmission and/or establishment of ancestral viral sequences in new hosts. This might be because ancestral viruses have an inherent transmission and/or establishment advantage, and/or because virus is preferentially stored in, and transmitted from, the genital tract as a consequence of compartmentalization.

A recent study has shown that although compartmentalization of the virus in the genital tract is apparent, this compartmentalized virus does not appear to be preferentially transmitted [35], suggesting that the mismatch in phylogenetic rates is not owing to compartmentalization. Moreover, if the mismatch in phylogenetic rates occurs because of compartmentalization, then we would only expect to see a mismatch in phylogenetic rates among viruses circulating in populations where the virus is sexually transmitted, and not in populations where the virus is transmitted intravenously. Contrary to this prediction, the rate of divergence of the virus circulating among IDUs tends to be even slower than the rate of evolution among populations where the virus is transmitted sexually [10], suggesting that preferential transmission owing to compartmentalization can be strongly ruled out as a mechanism for generating the mismatch.

If the virus has an inherent transmission and/or establishment advantage, however, then we speculate that we might well expect the rate of divergence to be slower in populations where transmission is intravenous rather than heterosexual. For a strain of HIV-1 to have a transmission and/or establishment advantage, it must be better than other strains at negotiating the mucosal barrier (if transmission is sexual), be better at gut homing, and/or have a faster growth rate during the first few days of infection. Ancestral virus is likely to have an advantage during this process because it will be very similar to the already successfully transmitted strain and will not have accumulated costly cytotoxic T-lymphocyte (CTL)-escape mutations. Larger inoculum sizes during high-dose rectal and intravenous transmission [34,36], the rapid dissemination of virus from rectal mucosa [37] and the lack of a mucosal barrier in intravenous transmission mean that it is more likely that ancestral virus will be successfully transmitted and therefore will be able to outgrow its competitors. During vaginal sexual transmission, stochastic effects are likely to be more important, making it more likely that ancestral virus is not given the opportunity to outgrow more contemporary strains.

In conclusion, the store and retrieve mechanism, with an inherent transmission and/or establishment advantage of ancestral virus is, of the mechanisms we considered, the only one leading to a mismatch in phylogenetic rates that alone is consistent with all of the available data. Such an inherent transmission and/or establishment advantage of ancestral virus is certainly plausible. During the course of infection, HIV-1 accumulates CTL-escape mutations that are likely to be deleterious to the virus when transferred into a new host [19,38,39]. Ancestral virus that has yet to accumulate these mutations will therefore have an advantage when infecting a recipient with a different HLA background to the donor. In addition, a number of characteristics of transmitted and founder viruses have recently been detected [38,40,41]. For example, transmitted viruses might have strong α4β7-reactivity compared with the circulating virus in the donor, which is potentially important because *α*4*β*7 is a marker for gut homing of CD4^+^ T cells [42]. It has also been suggested that the propensity of viruses establishing new infections to use the CCR5 coreceptor for host-cell entry is evidence of a transmission bias, since during the course of infection viruses typically evolve to use the CXCR4 coreceptor [1,11,43,44]. However, this conclusion has recently been challenged; the observed bias might simply be because few donors harbour X4 virus, and if they do the X4 variants tend to be at a relatively low frequency [45].

It is of course possible that the mechanisms described earlier act together to generate the mismatches we see, and we do not rule that out, but given the evidence currently available, the most parsimonious explanation is store and retrieve. Having discussed the conceptual model's qualitative ability to reproduce the data, we now test the concept further by quantitatively comparing the predictions of a mathematical formation of the store and retrieve model with the data reported in table 1. Our main goal is to establish whether the store and retrieve mechanism with an inherent transmission advantage can generate a rate mismatch of sufficient magnitude to explain the data given realistic parameter values.

## 4. A quantitative model of store and retrieve

We have created a mathematical model to explore the impact of the store and retrieve mechanism on evolutionary rates. We start from the by now standard model of within-host viral dynamics [46], but modify the model to enable us to keep track of the number of generations, *i*, a virus is removed from the founding strain (see also Kelly *et al*. [24]). We use a basic assumption that the rate of evolution along a lineage is proportional to the rate of replication along the lineage. This is true for neutral or nearly neutral mutations [47], but also for selective mutations since we are explicitly ruling out stage-specific selection from this model. As a consequence, viral lineages that have undergone fewer rounds of replication because the host was infected will be more similar to the infecting ancestral strain than viral lineages that have undergone more rounds of replication. Because most mutations occur at the reverse transcription stage, we assume that a virus advances one generation the moment it infects a cell. For brevity, we call strains that are removed by more generations from the infecting strain ‘more evolved’ than strains that are removed by fewer generations.

Our model follows the numbers of three host-cell types: active CD4^+^ T cells (susceptible, *S*, or infected, *I_i_*), latently infected memory CD4^+^ T cells (*L_i_*) and macrophage (susceptible, *M*, or infected, *X_i_*). The subscript *i* indicates cells infected by *i*th generation virus. In addition, the model tracks the number *i*th generation virus, *V_i_*.

For a full list of parameters and variables see table 2.4.1
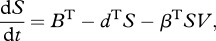
4.2
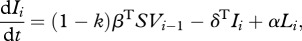
4.3
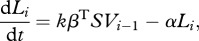
4.4
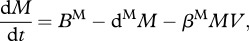
4.5
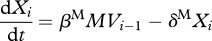
4.6
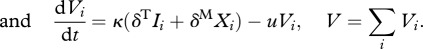

**Table 2.** Parameters and variables used for the within-host model. (All rates given per day. See main text for supporting references.)

*S*(*t*)	susceptible T cells	
*I_i_*(*t*)	activated T cells infected with virus generation *i*	
*L_i_*(*t*)	latent T cells infected with virus generation *i*	
*M*(*t*)	susceptible macrophage cells	
*X_i_*(*t*)	macrophage cells infected with virus generation *i*	
*V_i_*(*t*)	virus generation *i*	
*B*^T^	production rate of susceptible T cells	5 × 10^6^
*B*^M^	production rate of susceptible macrophage cells	5 × 10^4^
*d*^T^	death rate of susceptible T cells	0.5
*d*^M^	death rate of susceptible macrophage cells	0.05
*β*^T^	infection rate of T cells	1 × 10^−7^
*β*^M^	infection rate of macrophage cells	1 × 10^−7^
*δ*^T^	death rate of infected T cells	1
*δ*^M^	death rate of infected macrophage cells	0.1
*α*	activation rate of latent T cells	0.001
*k*	probability that infected cells enter latent stage	0 or 0.001
*κ*	viral growth rate	100
*u*	viral death rate	5

Here, we assume that the generation time of the virus when infecting active CD4^+^ T cells is 1 day (*δ*^T^ = 1; [27,46]) and when infecting macrophage is 10 days (*δ*^M^ = 0.1; [27]). The production rate of susceptible CD4^+^ T cells (*B*^T^) is 5 × 10^6^ and susceptible macrophage (*B*^M^) is 5 × 10^4^. CD4^+^ T cells and macrophage are assumed to have the same infection rate (*β*^T^ = *β*^M^ = 1 × 10^−7^) and the same viral growth rate when infected (*κ* = 100), consistent with the observation that about 10 per cent of virus produced in the body are derived from macrophage [27]. We assume that the probability that an infected CD4^+^ T cell enters the latent phase, *k*, is 0.001 and that the activation rate of latently infected memory CD4^+^ T cells, *α*, is 0.001 [28,29]. We also consider the case where *k* = 0; that is, where virus is not stored in memory CD4^+^ T cells.

**Figure 1.**
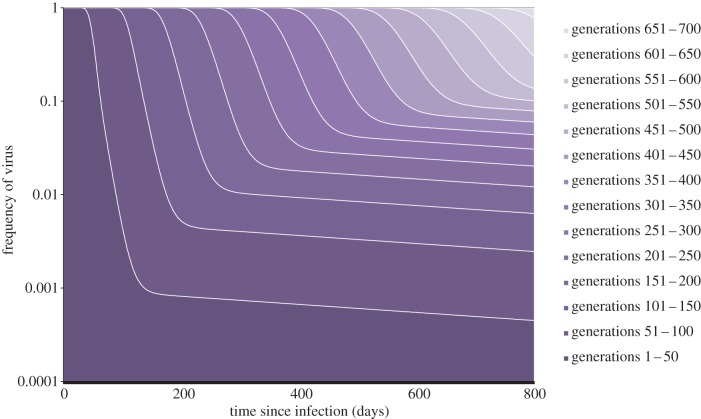
A stacked plot of viral generation frequencies within-host. The plot shows the proportion of free viruses within the host that have undergone 1–50, 51–100, … ,601–650 rounds of replication during the first 800 days of infection, for the case where *k* = 0.001. Dark shading indicates less evolved virus, and light shading more evolved virus.

**Figure 2.**
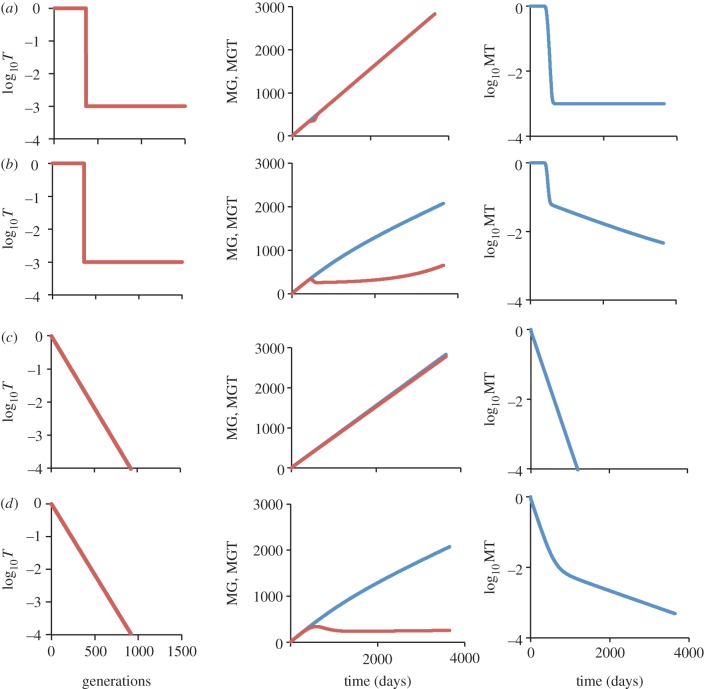
Mean number of generations and mean transmissibility in the virus population for two different patterns of loss of transmissibility. The first column shows the assumed decline in transmissibility (T) of the virus as it becomes more evolved (i.e. as the number of generations that the virus is removed from the founder strain increases). The second and third columns show the model output: the second column shows the mean number of generations the viral population has gone through in the host (MG, blue), and the mean number of generations in the transmitted virus (MGT, red), as a function of time since infection. The third column shows the mean transmissibility (MT) of the viral population as a function of time since infection. (*a*) Step function decline in transmissibility (*T* =1 if *i* < 365 else *T* = 0.001), no infected latent cells (*k* = 0). (*b*) Step function in transmissibility (*T* = 1 if *i* < 365 else *T* = 0.001), including infected latent cells (*k* = 0.001). (*c*) Exponential decline in transmissibility (*T* = e^−0.01*i*^), no infected latent cells (*k* = 0). (*d*) Exponential decline in transmissibility (*T* = e^−0.01*i*^), including infected latent cells (*k* = 0.001).

As a consequence of the virus circulating through different host-cell types, we see increasing heterogeneity in the amount of evolution circulating viral strains have undergone as infection progresses (figure 1). For this heterogeneity to result in the observed rate mismatch, ancestral virus must have a transmission advantage. In other words, as the virus evolves it should become less transmissible. Here, we consider two functions describing the pattern of loss of transmissibility, *T_i_*: step function (*T_i_* = 1 if *i* < 365 else *T* = 0.001; figure 2*a,b*); and exponential decline (*T_i_* = e^−0.01*i*^; figure 2*c*,*d*).

Using these loss of transmissibility functions, we calculate the mean number of generations viruses circulating with the host have gone through since infection (MG(*t*)), the mean number of generations transmitted virus have gone though since infection (MGT(*t*)) and the mean transmissibility of the viral population (MT(*t*)), where the maximum transmissibility is 1 and where *t* is the time since infection:4.7
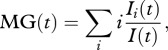
4.8
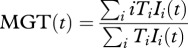
4.9
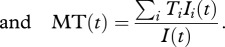


For the store and retrieve model to explain a slower rate of divergence at the between-host level than at the within-host level, the transmitted viral population must be less evolved than the general viral population circulating within the host. We find that when there is no storage of virus in memory CD4^+^ T cells there is very little difference between the circulating within-host population and the transmitted population (figure 2*a*,*c*). However, once we include storage the difference between the two populations becomes appreciable, reaching factors of four or more (figure 2*b*,*d*). We also see a concomitant drop in transmissibility for the viral population as a whole, which is plausible given the large drop in empirical estimates of infectiousness following acute infection [48].

We next calculated the mean number of generations accumulated by our model virus population per year at the within- and between-host levels (table 3) given the probabilities of transmission of the virus by stage of infection that were determined by Hollingsworth *et al.* [48]. The Hollingsworth *et al.* results were derived from Rakai cohort data involving HIV-1 heterosexual sero-discordant couples [49] under the extreme assumptions of either serial monogamy or random mixing. As expected, in our model, the virus population accumulates mutations at a similar rate at the within- and between-host levels when there is no storage of the virus in memory CD4^+^ T cells. However, when storage of the virus is allowed, we see a threefold difference in the rate of accumulation of viral generations when transmissibility declines in a step-like fashion, and a sixfold difference when transmissibility declines exponentially (table 3). The results are very similar regardless of whether we consider serial monogamy or random mixing.

**Table 3.** Mean number of generations accumulated within the viral population, per year, at the within- and between-host levels. (Data on duration of stages of infection and probability of transmission by stage of infection are from Hollingsworth *et al*. [48]. Duration of stages: primary, 0.24 years; chronic, 8.38 years; AIDS, 0.75 years.)

	within-host^a^	between-host (serial monogamy)^b^	between-host (random mixing)^c^
step decline			
no infected latent cells	282	284	285
infected latent cells	211	68	68
exponential decline			
no infected latent cells	282	280	281
infected latent cells	211	47	44

^a^Calculated during the chronic stage of infection.

^b^This is calculated as 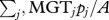, where MGT*_j_* is the average MGT during infection stage *j*, *p_j_* is the probability a new infection comes from a donor in infection stage *j* (primary, 0.09; chronic, 0.71, AIDS, 0.20) and *A* is the average time between transmission events (4.96 years).

^c^Calculated as for serial monogamy, but with values for *p_j_*: primary, 0.31; chronic, 0.42; AIDS 0.27. *A* = 4.33 years.

These results demonstrate that the storage of HIV-1 in long-lived memory CD4^+^ T cells, followed by preferential transmission of ancestral virus is a plausible mechanism leading to the observed mismatch in phylogenetic rates. Needless to say, in reality, the storage of HIV-1 is far more complicated than modelled here and the loss of transmissibility of viruses is unlikely to follow a simple step function or exponential decline; the function cannot be resolved without data. However, the model we have presented can be considered a proof of principle of the concept.

## 5. Discussion

In the past few years, data have emerged clearly showing that HIV-1 evolves much faster within hosts than it does at the epidemic level. Our aim here was to review published data to collate information on the magnitude of the mismatch, and then to systematically identify and evaluate the mechanisms that might cause this mismatch in rates of divergence. Given the available evidence, we argue that the storage of HIV-1 in very long-lived memory CD4^+^ T cells, followed by retrieval and preferential transmission of this stored virus, is the major factor contributing to the mismatch in rates of divergence at different levels of biological organization. We call this mechanism ‘store and retrieve’.

It is worth noting that within- and between-host rates of evolution have only been compared for *env*. However, there is good reason to believe that *env* might behave differently to other regions of the HIV-1 genome since it is under much stronger immunological pressure and therefore under strong directional selection. Areas of the genome where selection is more likely to be purifying or neutral would be expected to show different patterns of within- and between-host rates of evolution, depending on the mechanism driving the mismatch in rates of divergence. A preliminary analysis of whole genomes has shown that the mismatch is in fact higher in *env* than in other genes, but persists throughout the genome, indicating perhaps that unlike other viral genes, *env* is affected by both ‘adapt and revert’ and ‘store and retrieve’ dynamics simultaneously (Samuel Alizon 2011, personal communication).

Our study establishes the importance of acute infection in determining the evolutionary course of the HIV-1 pandemic: the viruses present in acute infection are stored and then preferentially transmitted. A major consequence of this observation is that what happens after acute infection, in the extreme case where only viruses stored during acute infection are transmitted, could be considered an evolutionary dead end at the population level (while remaining important for pathogenesis). This observation needs to be integrated into our thinking if we are to accurately model population level evolution of immune escape, drug resistance and virulence in HIV-1. Specifically, mutations selected for in early infection would be more likely to be transmitted than those selected for later. This could help explain the slow spread of many drug resistance mutations to date [50], even when we take into account the cost of resistance, except within clusters of individuals infected during acute infection [51]. Conversely, we also predict that pre-exposure prophylaxis will have a worse profile of resistance than otherwise expected [52]. In addition, early infection viruses should be the major target for vaccine design, because these are the viral stains primarily involved in transmission.
